# Novel Type IIS-Based Library Assembly Technique for Developing Nanobodies Targeting IPNv VP2 Protein

**DOI:** 10.3390/ijms26199350

**Published:** 2025-09-25

**Authors:** Camila Pino-Belmar, Johanna Himelreichs, Camila Deride, Tamara Matute, Isaac Nuñez, Severine Cazaux, Fernan Federici, Karen Moreno-Mendieta, Genaro Soto-Rauch, Joaquín Castro, Valentina Frenkel, Joi-Hui Ho, David Ascencios, Daniel Sanhueza Teneo, José Munizaga, Denise Haussmann, Alejandro Rojas-Fernandez, Jaime Figueroa Valverde, Guillermo Valenzuela-Nieto

**Affiliations:** 1 Institute of Biochemistry and Microbiology, Faculty of Sciences, Universidad Austral de Chile, Valdivia 5110566, Chile; 2 Institute of Medicine, Faculty of Medicine, Universidad Austral de Chile, Valdivia 5110566, Chile; 3 Agencia Nacional de Investigación y Desarrollo—Millennium Science Initiative Program—Millennium Institute for Integrative Biology (iBio), Schools of Engineering, Medicine and Biological Sciences, Pontificia Universidad Católica de Chile, Santiago 8331150, Chile; 4 Institute for Biological and Medical Engineering, Schools of Engineering, Medicine and Biological Sciences, Pontificia Universidad Católica de Chile, Santiago 8331150, Chile; 5 Bain & Company, Santiago 7550268, Chile; 6 Institute of Immunology and Parasitology, Faculty of Medicine, Universidad Austral de Chile, Valdivia 5110566, Chile; 7 Biochemistry School, Faculty of Sciences, Universidad Austral de Chile, Valdivia 5110566, Chile; 8 Facultad de Ciencias para el Cuidado de la Salud, Universidad San Sebastián, Valdivia 5090000, Chile; 9 Departamento de Ciencias Básicas, Facultad de Ciencias, Universidad Santo Tomás, Valdivia 5110566, Chile; 10 Berking Biotechnology, Valdivia 5090000, Chile; 11 Interdisciplinary Center for Aquaculture Research (INCAR), Concepción 4030000, Chile

**Keywords:** IPNv, single domain antibodies, VP2, Type IIS-based assembly methods

## Abstract

The development of effective tools to combat viral diseases remains a major challenge for the aquaculture industry. Infectious pancreatic necrosis virus (IPNv) is one of the most devastating pathogens affecting salmonids, leading to high mortality rates and substantial economic losses worldwide. Here, we present a novel nanobody discovery pipeline based on a Type IIS restriction enzyme-driven library assembly method that enables the rapid generation of highly diverse nanobody repertoires. This streamlined approach not only shortens the time required for nanobody identification but also offers remarkable adaptability, allowing its application to virtually any protein target, including antigens from aquaculture pathogens and beyond. By integrating this strategy with density gradient–based enrichment and high-throughput screening, we successfully identified and validated a nanobody against the VP2 protein of IPNv, a key structural component essential for viral infectivity. These findings highlight the potential of this platform both as a versatile methodological advance in antibody engineering and as a practical foundation for developing innovative diagnostic and therapeutic tools. Ultimately, nanobodies generated through this pipeline could play a pivotal role in improving disease management and enhancing sustainability in aquaculture.

## 1. Introduction

Infectious pancreatic necrosis virus (IPNv) poses a critical and persistent challenge for the global salmon farming industry. It is a double-stranded RNA virus belonging to the family *Birnaviridae*, genus *Aquabirnavirus* [1]. The genome of IPNv consists of two segments of double-stranded RNA, packaged within a non-enveloped icosahedral capsid approximately 60 nm in diameter. Segment A encodes the VP2, VP3, VP4, and VP5 proteins, while segment B encodes an RNA polymerase (VP1). VP2 and VP3 are the two main structural proteins of this virus [1,2,3]. VP2 is a 54 kDa protein that is the primary component of the capsid by number of units and is responsible for binding to the host cell [1,4,5]. It is composed of three domains: a central domain (S), a base located towards the interior of the viral particle, and an outward-facing spike or projection domain (P). The spikes, organized as VP2 trimers around an axis, contain the primary viral antigenic sites, the cell specificity epitope, and some virulence determinants. Since the 1980s, neutralizing epitopes in VP2 have been described, primarily located in an internal region of VP2 between amino acids 206 and 350, which is recognized by the monoclonal antibody (MAb) 17/82. This antibody has been reported to neutralize viral infection [2,3,6]. The host cell binding site is in the P domain of VP2, at the top of the spike; however, certain amino acids located in the groove of the spike, near the S domain, are also important for cell binding. It has been suggested that the former are responsible for recognition and binding, while the latter are involved in the internalization process [5].

IPNv primarily affects salmon during their freshwater stage, leading to extremely high mortality rates, reaching up to 90% in severe cases, especially among fry and juveniles [7]. High mortality not only disrupts the later development of fish in seawater but also has a significant economic impact. Surviving fish serve as viral reservoirs, thereby facilitating their spread in high-density aquaculture environments, such as recirculating aquaculture systems (RAS) and open-sea cages [8]. Additionally, the virus can remain active in the environment, including in soil and aquaculture infrastructure, for several weeks, further complicating its control [9]. The economic losses associated with IPNv extend beyond direct mortality; the virus also induces epigenetic modifications that predispose fish to subsequent viral and bacterial infections during their seawater growth phase, significantly increasing losses by affecting both the quality and quantity of the final product [10].

Despite efforts to control IPNv through vaccines and selective breeding programs, there has been an observed increase in outbreak frequency, even in salmon populations genetically selected for IPNv resistance. Recent outbreaks among genetically resistant salmon in Chile, Scotland, and Norway suggest that the virus is developing mutations, particularly in the VP2 protein, enabling it to evade host defenses [11,12]. These outbreaks may be due to mutations in the hypervariable region (HPR) of VP2, which warrants further investigation [11].

Emerging IPNv strains, characterized by mutations in the VP2 protein, have shown adaptability to different salmonid species and the ability to overcome immune barriers. This underscores the urgent need for adaptable molecular tools that can be deployed quickly, with immunotherapy presenting a promising advanced tool against viral variants. However, the high costs of monoclonal antibodies limit their feasibility for aquaculture. This is where a promising alternative emerges: the use of nanobodies. Derived from single-chain antibodies in camelids, nanobodies offer significant advantages over conventional therapies. Their small size and unique structure allow them to bind to viral epitopes that are inaccessible to other antibodies, resulting in high specificity and neutralizing capacity. Additionally, nanobodies are more stable and easier to produce than traditional antibodies, making them a cost-effective tool for scenarios that require affordable solutions. The use of nanobodies offers a flexible and effective solution to address the constant emergence of new viral variants, making them a promising therapeutic tool to improve salmon health and reduce associated economic losses. However, their application in aquaculture remains scarce, and bridging this gap represents both a significant scientific opportunity and an urgent need for the sector [13,14].

However, one of the main bottlenecks in nanobody development lies in the generation of high-quality, diverse libraries. This is typically achieved using traditional cloning with restriction enzymes, following protocols that are often time-intensive to ensure optimal library quality. In this study, we explored alternative assembly methods to improve the efficiency of this process. Due to their capacity to generate sequence variants in a single reaction, assembly techniques based on Type IIS enzymes, like the Golden Gate method and its derivatives, have proven highly effective for large-scale DNA library construction [15,16,17]. These techniques enable the simultaneous assembly of multiple DNA fragments by designing unique overhangs, facilitating the seamless and efficient creation of highly diverse sequence libraries [18,19]. This approach offers a significant advantage over traditional cloning methods, which often require multiple ligation and transformation steps for each DNA fragment, a process that is labor-intensive and error-prone [17].

Type IIS-based assembly methods, such as the Universal Loop (uLoop), reduce errors and accelerate the cloning process by allowing multiple fragments to be assembled in a single-step reaction [20,21]. Furthermore, these techniques can be designed to create new restriction sites post-ligation, facilitating additional rounds of cloning in different vectors for expanded applications. Type IIS enzyme-based methods enable high-throughput DNA library generation, making them invaluable tools for applications like protein engineering, pathway optimization, and synthetic biology, where diverse DNA libraries are essential for exploring genetic variability efficiently [16,17,19,20,22,23,24].

This study presents an innovative nanobody library assembly technique based on the use of Type IIS restriction enzymes, applied to identify nanobodies targeting the VP2 protein of IPNv.

## 2. Results

### 2.1. Recombinant VP2 Expression and Purification

**Figure 1:**
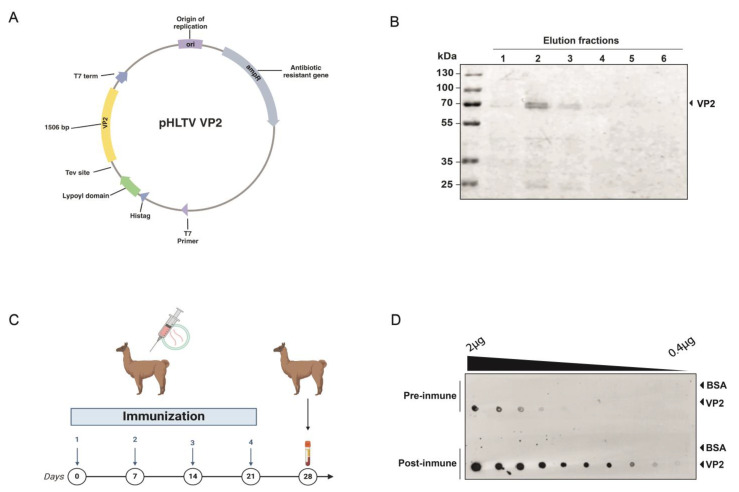
Recombinant VP2 protein production and immunization. (**A**) Scheme of the vector generated for the expression and purification of the IPNv VP2 protein in *E. coli*. The coding sequence for the protein, totaling 1506 bp, is highlighted in yellow. (**B**) SDS-Page to ensure protein integrity of recombinant VP2 protein before immunization. (**C**) Diagram of the alpaca immunization process. (**D**) Evaluation of the alpaca’s immune response by dot blot. The image shows the reaction to decreasing amounts of recombinant IPNv VP2 protein and bovine serum albumin (negative control) using a preimmunization control, and post-immunization with VP2, using alpaca serum as a primary antibody source, followed by an anti-camelid IgG-HRP secondary antibody.

A 1.5 kb fragment encoding VP2 (GenBank accession number AY379740.1) was successfully cloned into the pHLTV vector using Gibson assembly, enabling expression with an N-terminal 6xHis tag and a dihydrolipoamide acetyltransferase (E2) lipoyl domain to enhance solubility (Figure 1A) [25,26]. The resulting construct was transformed into *E. coli* strain BL21, followed by induction and purification using Nickel affinity chromatography. This process yielded 10 mL of recombinant VP2 protein at a concentration of 0.5 mg/mL, which was subjected to SDS-PAGE (Figure 1B) to examine the target protein expression. A band near 70 kDa was detected, consistent with the expected size of 6xHis-Lipoyl domain-Tev site-VP2 fusion protein (66.571 kDa).

### 2.2. Alpaca Immunization

A male alpaca was immunized four times over a 28-day period with 100 μg of recombinant VP2 protein combined with an adjuvant (Figure 1C) [27]. The animal health was monitored throughout the study via clinical examinations, hematological analysis, and serum biochemistry. The immune response in the alpaca’s serum was assessed using Dot blot analysis as a rapid qualitative method, immobilizing the antigen onto a nitrocellulose membrane and using the alpaca serum as the source of primary antibodies. A significant increase in antigen-specific IgG antibodies in the alpaca’s serum was observed, resulting in a post-immune serum capable of detecting even picograms of very small amounts of the protein, such as those found in the tenth dilution, approximately 0.4 ng (Figure 1D). Additionally, pre- and post-immunization sera were compared as primary antibodies to detect the viral protein in anterior kidney imprints of *Salmo salar* positive for IPNv infection. Post-immune serum yielded a markedly stronger signal compared with both pre-immune serum and post-immune serum tested on IPNv-negative samples (Supplementary Figure S1).

### 2.3. Library Preparation

**Figure 2:**
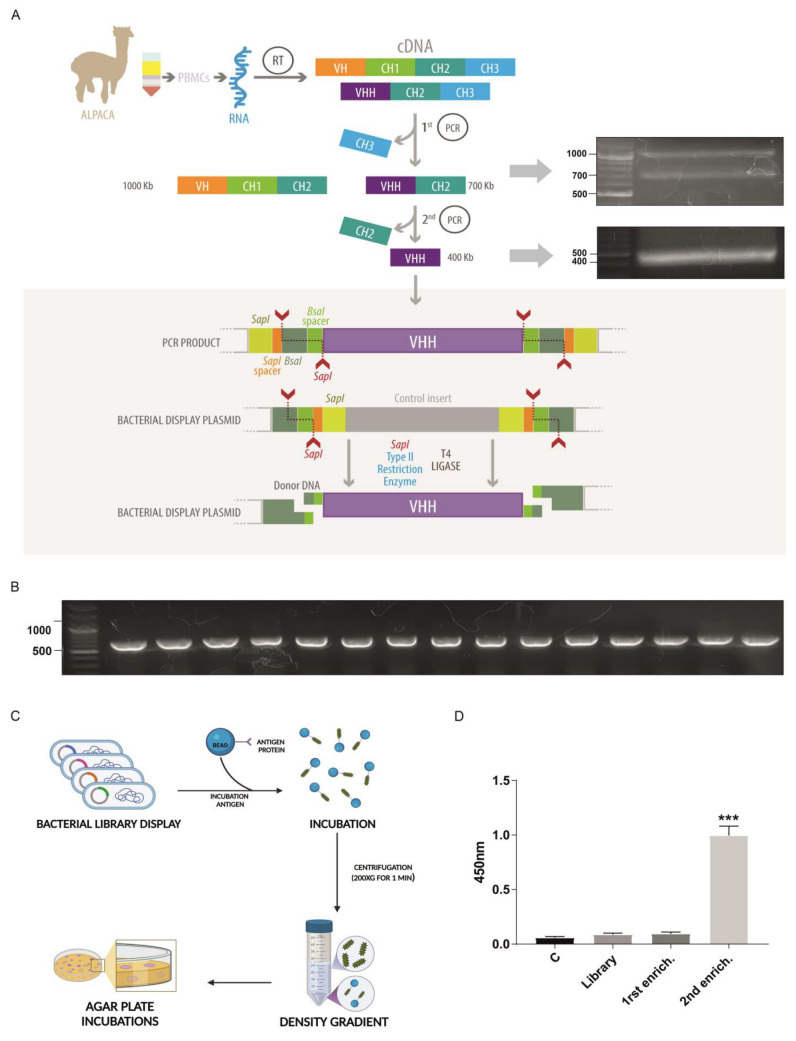
Novel type IIS restriction enzyme-based method for bacterial display library generation. (**A**) Overview of the library construction process starting from RNA extraction from alpaca PBMCs, followed by reverse transcription to generate cDNA, and amplification of the VHH coding region through two rounds of PCR. The side panels display agarose gel electrophoresis of the PCR products obtained at each step during the library generation for VP2. The amplified VHH fragment is inserted into a bacterial display plasmid using *Sap*I and Type IIS restriction sites, allowing seamless assembly of the library through a one-tube digestion-ligation cyclic reaction that also generates a new restriction site, *Bsa*I. (**B**) Gel electrophoresis results confirming successful insertion and uniform size of VHH fragments in the bacterial display library clones. (**A**,**B**) Electrophoresis us TAE 1× 1% agarose gels using Generuler 100 bp plus standard (Thermo). (**C**) Schematic of the density gradient enrichment protocol, including incubation with beads coated in target antigens (VP2 recombinant protein), followed by a Ficoll gradient-based separation. (**D**) ELISA assay of VP2 protein using total protein extract from library and the following density gradient enrichment rounds. This shows a significant signal increase after the second round, in which approximately 300 clones were recovered and used in subsequent screening. *n* = 3. Statistical *t*-test, *** *p* ≤ 0.001 to control. Illustration (a) by Felipe G. Serrano BSc., MSc Scientific illustrator.

The pNeae2 and pHEN6 plasmids were successfully modified by mutagenic PCR to replace the *Sfi*I and *Not*I restriction sites with *Sap*I and *Bsa*I, following the cloning strategy described by Pollak and collaborators [21]. The resulting plasmids, named pNeae2.1 and pHen6.1, enabled improved cloning compatibility for subsequent experiments. Our method for nanobody isolation builds on the bacterial display system previously described by Salema and collaborators [28], which has been successfully utilized by our team in earlier studies [27]. We implemented a novel procedure for library generation, utilizing Type IIs restriction enzymes that recognize specific DNA sequences but cut, at distinct sites, a defined number of base pairs away based on the uLoop system [21]. This approach involves an innovative strategy where cutting sites are arranged on both the insert and the vector in such a manner that upon ligation, the *Sap*I recognition site is removed, enabling the reaction to proceed, all in one tube, in alternating cycles of digestion and ligation at 37 °C and 16 °C, respectively. As the *Sap*I sites on the vector are progressively eliminated with each ligation of the VHH insert (the coding region for the nanobody), the reaction increasingly favors the formation of vector-insert constructs, dramatically reducing the time needed to generate a bacterial display library (Figure 2A). Ligation also assembles a new restriction site for *Bsa*I, useful for further cloning steps. The detailed protocol for library assembly is provided in Section 4. Briefly, it involves two rounds of PCR: the first round amplifies the VH, CH1, and CH2 regions of heavy-chain antibodies, as well as the VHH and CH2 regions of single-chain antibodies, yielding bands of approximately 1 kb and 700 bp, respectively (Figure 2A first panel). These bands are separated electrophoretically, retaining only the 700 bp band for use as a template in a subsequent PCR reaction. This second PCR specifically amplifies the VHH coding region for nanobodies, adding *Sap*I sites and adaptors required for subsequent ligation into the pNeae2.1 vector, obtaining a PCR product around 400 bp (Figure 2A, second panel). Thus, a bacterial display library was constructed, with a complexity of 1 × 10^6^ independent clones via electroporation of the *E. coli* DH10B-T1R strain. Random clones were screened by PCR to verify insert size, with 100% of clones showing inserts of the expected size for a nanobody. This was further confirmed by sequencing, which additionally demonstrated that 100% of the clones corresponded to nanobodies and were in the correct reading frame, with the *Bsa*I site in the right position (Figure 2D).

We used a novel procedure for Nanobody selection using a simple Ficoll density gradient, previously used by our group with great success [27]. This takes advantage of the bacterial display system, in which each bacterium in the library expresses a single Nanobody clone [28]. *E. coli* bacteria express intimin-Nanobody fusion proteins anchored in the outer membrane, exposing the functional Nanobody to the extracellular space for antigen recognition. Bacteria expressing specific nanobodies on their surface were incubated with Sepharose beads covalently coated with recombinant VP2, the antigen of interest, and migrated to the bottom of the Ficoll density gradient, while unbound bacteria remained in the upper fraction. Two rounds of enrichment were performed using this protocol, and the ability of the resulting clone pools from each round to recognize recombinant VP2 protein was assessed by ELISA. These enriched pools were also compared to the complete library and a control lacking nanobodies. A significant increase in VP2 detection by ELISA was observed for the clone pool obtained after the second round of density gradient enrichment, making this pool the optimal choice for identifying specific clones (Figure 2D).

### 2.4. Nanobody Candidate Selection

**Figure 3:**
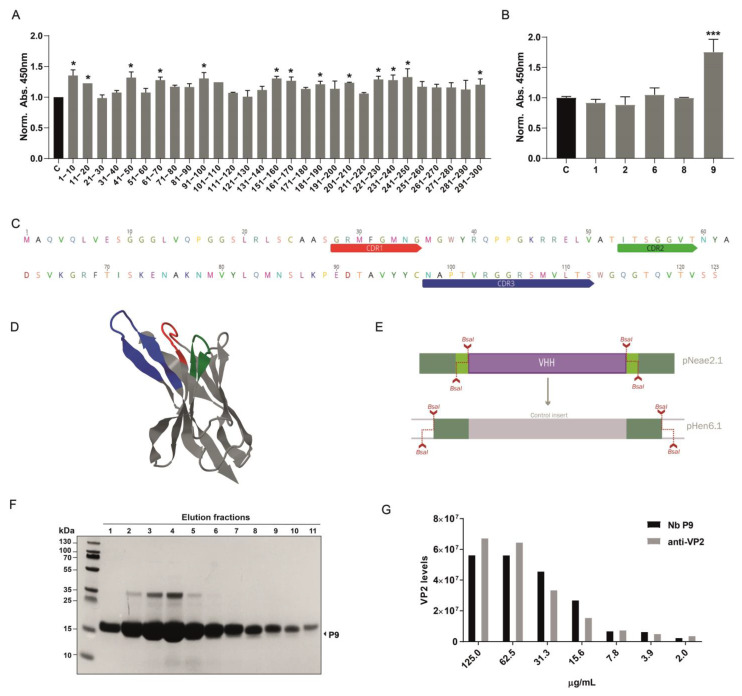
Identification and expression of a nanobody against IPNv VP2. (**A**) Initial screening of nanobody pools (30 pools, each containing 10 clones) was performed using ELISA on VP2-coated plates and direct total protein extracts of clones as primary antibodies. Mouse anti-myc (1:3000) followed by anti-mouse-HRP was used for detection. Among these, 13 pools displayed significant binding to recombinant VP2 protein. Graphs depict the normalized to control mean of 450 nm absorbance, *n* = 3. Statistical *t*-test, * *p* ≤ 0.05 to control (**B**) Example of secondary screening through ELISA of individual clones from 1–10 positive pool, in which we identified clone P9 as a positive binder for VP2. Statistical *t*-test, *** *p* ≤ 0.001 to control. (**C**) Sequence analysis of clone P9, using IMGT/V-QUEST identified the complementary determining regions (CDRs), crucial for binding specificity. (**D**) Alphafold structural prediction based on P9 sequence, CDRs are depicted in the same color scheme as in C. (**E**) Diagram of the subcloning strategy used to enable periplasmic expression of the isolated P9 clone. This strategy leverages the *Bsa*I site generated during library cloning, allowing a rapid transition to a new vector, in this case, pHEN6.1. (**F**) SDS-PAGE shows the purification of the P9 clone from the *E. coli* periplasm. (**G**) Quantification of VP2 levels (in arbitrary units) detected by dot blot analysis using purified P9 and commercial anti-VP2 antibody (Ango, clone 1B3/E10).

Following nanobody selection using the density gradient protocol, approximately 300 colonies were obtained on LB-agar plates from the Sepharose-antigen-coated fraction. We optimized conditions to extract intimin-Nanobody fusions from the bacterial outer membrane and used these protein extracts directly to assess binding to the VP2 protein by ELISA. Initially, 30 pools of 10 clones each were analyzed by incubating VP2-coated ELISA plates with bacterial extracts containing nanobodies. Sequential incubation with a mouse anti-myc antibody, followed by an anti-mouse HRP-conjugate, revealed that 13 pools showed significant recognition of recombinant VP2. A secondary screening of individual clones was then performed (Figure 3A). For example, in the pool containing clones 1–10, clone P9 demonstrated a strong ability to recognize recombinant VP2 in the ELISA assay (Figure 3B). The selected clone was sequenced, and CDR regions were identified using IMGT/V-QUEST [29] (Figure 3C). Additionally, the structure was predicted using AlphaFold, highlighting the CDRs (Figure 3D and Supplementary Figure S2). The P9 clone was subsequently cloned into the pHen6.1 vector for periplasmic bacterial expression, using *Bsa*I sites and a similar assembly strategy as the one used for library construction (Figure 3E). Large amounts of recombinant P9 nanobody were obtained, around 20 mg per liter of culture. (Figure 3F). The purified P9 nanobody was first validated as a primary antibody by dot blot. Serial dilutions of IPNv total protein extract were spotted as antigen, and signals were compared with those obtained using a commercial anti-VP2 antibody. Quantitative analysis showed that, across the entire concentration range tested, Nb P9 yielded higher signal intensities, indicating greater sensitivity and a lower apparent limit of detection. Although signal intensity decreased with antigen concentration, it remained detectable even at the lowest dilutions (Figure 3G).

### 2.5. Molecular Interaction Prediction

To explore the putative molecular interaction between nanobody P9 and the VP2 protein, we conducted molecular docking guided by five complementary metrics, ipTM, ipSAE, pDockQ, pDockQ2, and LIS. As validation, the same pipeline was applied to a positive interaction control (CD2BP2–CD2; UniProt IDs O95400 and P06729) and a negative interaction control (CD2BP2–PSMD4; UniProt IDs O95400 and P55036). The positive control complex yielded consistently high interface-confidence scores (ipTM = 0.22; ipSAE = 0.123; pDockQ = 0.160; pDockQ2 = 0.049; LIS = 0.245) when predicted with AlphaFold 3, whereas the negative control showed markedly lower or null values across all metrics (ipTM = 0.08; ipSAE = 0.000; pDockQ/pDockQ2 = 0; LIS = 0). These results confirmed that the chosen thresholds discriminate bona fide interactors from non-interactors.

**Figure 4:**
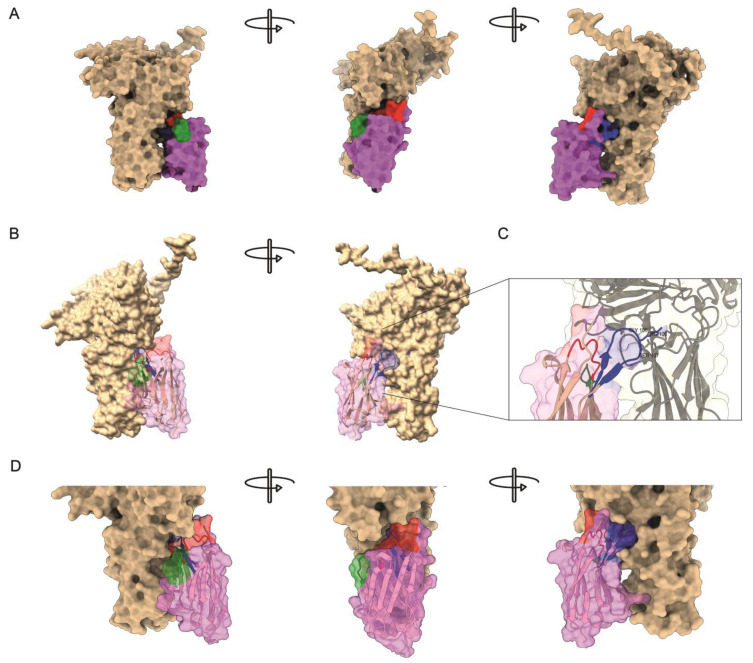
Predicted molecular interaction between P9 nanobody and VP2. (**A**) Structural representation of the interaction model of the VP2–P9 complex. VP2 is shown in tan, whereas the P9 nanobody appears in magenta; its CDRs are colored as in Figure 3 (CDR1 red, CDR2 green, CDR3 blue). (**B**) Same model with the P9 surface rendered semitransparent to expose the underlying ribbon trace. (**C**) Close-up of the primary binding interface. CDR3 residues Gly105, Arg106, and Ser107 project into a solvent-exposed cavity of the VP2 projection domain. Side chains within 4 Å are displayed as sticks, and hydrogen bonds or salt bridges are indicated by dashed lines. (**D**) Close-up of the interaction model with the P9 rendered semitransparent; here, an additional interaction mediated by residue Arg46, from framework region (magenta), can be observed.

For the VP2–P9 model, AlphaFold 3 produced an ipTM of 0.12 and an ipSAE of 0.0026, indicating modest global confidence but a very low proportion of high-certainty interface residues. In contrast, pDockQ rose to 0.243, exceeding the empirical 0.23 threshold often associated with real interactions, while pDockQ2 and LIS remained low (0.009 and 0.000, respectively). The Boltz-1 workflow reproduced this weak-to-moderate trend (ipTM = 0.11; ipSAE = 0.00) (Figure 4A).

ParaSurf detected discrete hotspots (probability > 0.5) on both VP2 and P9 that converge at a single narrow patch where the two chains approach each other. The 3D rendering reveals that any association between VP2 and P9 is limited to a small subset of residues, consistent with the low ipSAE but intermediate pDockQ values, grouped on a distinct interaction hotspot within the CDR3 loop of P9, centered on residues Gly105, Arg106, and Ser107, which protrude into a cavity on VP2, together with an additional contact around residue Arg46 (Figure 4B,C). These make contact with the 206–350 segment of the VP2 projection domain, previously identified as the epitope targeted by neutralizing antibodies such as mAb17/82 [2] (Supplementary Figure S3).

### 2.6. Nanobody Validation

**Figure 5:**
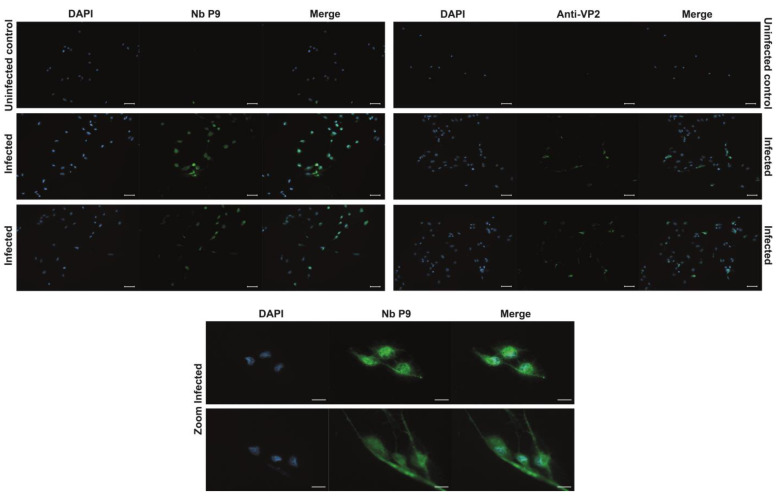
Immunofluorescence detection of IPNv in vitro infected SHK-1 Cells. Representative immunofluorescence micrographs of SHK-1 cells infected with infectious pancreatic necrosis virus (IPNV, strain VR-299). Viral antigens were labelled with the P9 nanobody (primary antibody, 1:1000 in 1 × PBS; stock concentration 1 mg mL^−1^). Bound nanobodies were detected with a mouse anti-myc tag monoclonal antibody (Cell Signaling; 1:3000), followed by an Alexa Fluor 488-conjugated goat anti-mouse IgG secondary antibody. Images obtained with a commercial anti-VP2 antibody (Anwo) are included for comparison in the right column. DAPI was used to counterstain the nuclei. The upper row shows mock-infected control cells; the two middle rows display infected cells; the bottom row presents higher-magnification views of infected cells to highlight viral antigen localization. Scale bars: 50 µm in the three upper rows and 20 µm in the two lower rows.

To evaluate the ability of the P9 nanobody to recognize IPNv in situ, SHK-1 cells were infected with the reference strain VR-299 and examined 48 h post-infection. Infected cells exhibited a strong, punctuate cytoplasmic and peri-nuclear fluorescence when labelled with P9 (Figure 5, left column and bottom rows). This signal was completely absent in mock-infected controls, confirming that background reactivity of the detection system was negligible. Importantly, the spatial distribution, intensity, and sub-cellular localization of the P9-derived fluorescence were indistinguishable from those obtained in parallel samples stained with a well-validated commercial anti-VP2 monoclonal antibody (Figure 5, right column). The concordance between the two staining patterns provides compelling evidence that P9 recognizes native VP2 in infected salmonid cells with high specificity, thereby validating its use as a diagnostic or research reagent for IPNv detection.

**Figure 6:**
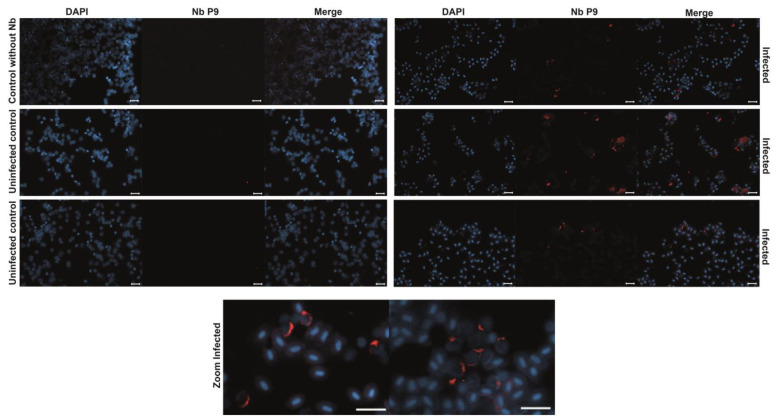
Immunofluorescence Detection of IPNv-Infected Cells in *Salmo salar*. Immunofluorescence staining of head kidney imprints from *Salmo salar* samples confirmed to be IPNv-positive (by qPCR) and uninfected controls. Cells were incubated with the purified P9 nanobody as the primary antibody (1:3000 from 1 mg/mL stock), followed by a mouse anti-Myc antibody and an Alexa 647-conjugated anti-mouse secondary antibody. DAPI was used to counterstain the nuclei. A distinct fluorescent signal is observed in IPNv-infected cells, indicative of specific recognition by the P9 nanobody, while uninfected control samples show no significant signal, supporting the diagnostic potential of P9 for IPNv detection in aquaculture settings. Bars = 20 µm.

Additionally, the purified P9 nanobody was used as a primary antibody in immunofluorescence assays with imprints of *Salmo salar* head kidney samples, positive (qPCR tested) for IPNv. We observed a characteristic fluorescence pattern, consistent with previous reports using commercial antibodies [30,31], with no signal detected in negative or uninfected controls (Figure 6). This suggests that the P9 nanobody specifically recognizes IPNv-infected samples (Figure 6). Thus, our experiment suggests the P9 nanobody is applicable as a tool for the direct diagnosis of infected cells by immunofluorescence.

## 3. Discussion

Nanobodies represent a promising tool for combating pathogens, highlighting the importance of enhancing and optimizing the processes by which they are generated [32,33,34,35,36,37]. This study introduces a novel and highly efficient method for generating nanobody libraries, leveraging Type IIS restriction enzyme-based assembly to accelerate the cloning process, based on the uLoop system [21]. This streamlined approach facilitated the rapid development of a bacterial display library, enabling efficient screening and selection of nanobodies. This method not only reduces the time required for library assembly, from around two weeks to two days, but also minimizes errors associated with traditional cloning techniques. Additionally, this method enables concatenated, multi-level cloning through an ingenious design in which the ligation of the first level generates a new restriction site that can be utilized in the subsequent cloning level, allowing for rapid transfer to other vectors [15,20,23,24,38]. The advantages of this technology are particularly valuable in scenarios where a swift response is critical, such as during infectious disease outbreaks, shortening the time for isolation and development of specific nanobodies.

Here, we have shown a successful example based on the immunization of IPNv VP2 protein. Subsequent rounds of enrichment by density gradient, using a cost-effective protocol previously developed by our group, enabled the generation of a panel of 300 nanobodies [27]. These nanobodies underwent secondary screening, allowing for the rapid identification of candidate clones. We report the example of clone P9, which was subsequently subcloned into a periplasmic expression vector using the *Bsa*I restriction site generated during library preparation. Thus, undoubtedly, coupling this novel and efficient library generation method with density gradient enrichment, alongside rapid high-throughput screening methods such as pooled clone ELISA or high-content microscopy, represents a significant advancement in accelerating the acquisition and identification of new nanobodies.

Our successful isolation and characterization of a nanobody (P9) with specificity for IPNv VP2 offers a contribution with the potential to pave the way for the adoption of these new technologies within the aquaculture industry. Given the significant economic impact of IPNv on the global salmon industry, this nanobody presents a promising diagnostic tool for detecting IPNv infections in fish tissues. The indistinguishable staining patterns obtained with P9 and the commercial anti-VP2 antibody [30,31], in both infected SHK-1 cells and qPCR-positive head-kidney imprints, underscore the P9 nanobody’s high specificity for native VP2 and highlight its promise as a scalable, cost-effective immunofluorescent reagent for routine IPNv diagnosis in aquaculture settings.

Furthermore, the structural interaction predictions yield valuable insights into this novel nanobody. Although certain models suggest that the overall binding affinity may be modest, the principal contact occurs between CDR3 and a segment of VP2’s projection domain, a region previously identified as a hotspot for neutralizing antibodies [2,39]. This finding underscores the dual potential of P9: in addition to its utility as a diagnostic tool, it may also serve as an effective neutralizing antibody candidate, an avenue worth exploring because of its relevance for the health and sustainability of aquaculture practices.

Moreover, the specificity and ease of production of nanobodies offer an alternative to monoclonal antibodies, which are often costly and labor-intensive to produce. Nanobodies, due to their smaller size and structural characteristics, allow for high stability and high binding affinity, making them more suitable for routine and large-scale diagnostic or therapeutic applications in the aquaculture industry [40]. The successful application of this approach to isolate a nanobody against IPNv VP2 could easily be adapted for other pathogens, broadening the impact of this methodology.

In conclusion, the nanobody library assembly technique presented here provides an innovative and efficient approach for generating high-quality nanobodies. This technology has significant potential to improve health management practices and support economic sustainability in aquaculture by addressing emerging threats, such as the appearance of new viral variants. Moreover, it is adaptable to a range of pathogens and various industrial applications.

## 4. Materials and Methods

### 4.1. Expression and Purification of VP2 Protein

The nucleotide sequence of the VP2 protein was obtained from GenBank (accession number AY379740.1), synthesized, and cloned into the pHLTV vector using Gibson assembly. Specific primers were designed to amplify the VP2 coding sequence (fwd: CTTCCAGGGTGGATCCATGAACACAAACAAGGCAACCGC, rev: AGCCGGATCAAGCTTCGGTCTTTGTAGCGCCCTCCTGCGGCC) and the vector backbone (fwd: GAGGGCGCTACAAAGACCGAAGCTTGATCCGGCTGCTAACAAAG, rev: TTGTTTGTGTTCATGGATCCACCCTGGAAGTACAGGTTTTC). To reduce the probability of aggregation via electrostatic repulsion between highly charged soluble polypeptides, the construct was assembled to express VP2 fused with a 6xHis tag and a Lipoyl domain (from dihydrolipoamide acetyltransferase). Thus, solubility was enhanced, and adequate time for correct folding was allowed. Additionally, these solubility enhancers might act as intramolecular chaperones by participating in native folding of the target proteins [25,26]. The resulting construct was then transformed into *E. coli* strain BL21. A pre-inoculum was prepared by adding 10 µL of glycerol stock from a previously transformed *E. coli* BL21 strain to 15 mL of liquid LB medium supplemented with ampicillin. This culture was incubated overnight at 37 °C with shaking. The next day, 100 mL of LB medium was inoculated with the overnight culture and incubated at 30 °C for 2.5 h with shaking. This culture was then used to inoculate 500 mL of LB medium, which was incubated at 37 °C with shaking for an additional 2.5 h. The bacteria were harvested by centrifugation at 3000× *g* for 15 min at 4 °C. The bacterial pellet was resuspended in 1 L of 2YT medium, incubated at 42 °C for 30 min with agitation, and then cooled to 16 °C for 10 min. The optical density at 600 nm (OD_600nm_) was measured to ensure a range between 0.6 and 0.8. Protein expression was induced by adding 10 µM IPTG, and the culture was incubated at 16 °C for 40 h with shaking. After induction, the bacteria were collected by centrifugation at 3000× *g* for 15 min at 4 °C. Induced bacterial pellets from 1 L culture were resuspended in 10 mL of lysis buffer (50 mM Tris (pH 7.5), 500 mM NaCl, 10 mM imidazole, 1 mM DTT, 1× protease inhibitor). Cells were lysed by sonication (50% pulse, 20 s on/off intervals for 5 min) and centrifuged at 21,000× *g* for 45 min at 4 °C. The supernatant was recovered and filtered through a 0.22 µm syringe filter. Protein purification was conducted using Ni-NTA affinity chromatography. The filtered lysate was applied to a Ni-NTA column containing 3 mL of pre-equilibrated agarose beads, compacted to a 2 mL bead bed. The column was washed with 20 mL of binding buffer (50 mM Tris (pH 7.5), 500 mM NaCl, 10 mM imidazole, 1 mM DTT), followed by 30 mL of wash buffer (50 mM Tris (pH 7.5), 500 mM NaCl, 30 mM imidazole, 1 mM DTT), and the target protein was eluted with 10 mL of elution buffer (50 mM Tris (pH 7,5), 500 mM NaCl, 150 mM imidazol, 1 mM DTT). Purified protein was verified by SDS-PAGE followed by Coomassie staining.

### 4.2. Alpaca Immunization

The alpaca immunization procedure followed the ethical guidelines of the Bioethics Committee of the Austral University of Chile (certifications 463/2022) and was carried out as previously described by [27]. A 5 mL blood sample was collected one day before immunization to obtain pre-immune serum. For the initial immunization (day 1), 100 μg of recombinant VP2 protein was used. The recombinant protein elution buffer was exchanged to PBS 1×, then mixed with 2 mL of Veterinary Vaccine Adjuvant (GERBU FAMA) and administered subcutaneously to a male alpaca (*Vicugna pacos*) at four different sites, delivering a total volume of 4 mL. Seven days post-immunization, a 5 mL blood sample was taken. On day 14, a second immunization was performed using 100 μg of VP2 protein. On day 15, a 120 mL blood sample was collected from the jugular vein into tubes containing 3.8% sodium citrate as an anticoagulant. This blood was diluted with an equal volume of HBSS medium (Gibco, Waltham, MA, USA) without calcium, and the mixture was aliquoted into 10 mL portions, each layered on 5 mL of Ficoll-Paque Premium (GE Healthcare) in 15 mL sterile Falcon tubes. After centrifugation (1200 rpm, 80 min, room temperature), the PBMC layer was carefully collected, washed twice with PBS by centrifugation (3500 rpm, 10 min), and resuspended in 4 mL of sterile PBS (Gibco). RNA extraction was performed using the RNeasy Mini Kit (Qiagen, Venlo, The Netherlands), and cDNA synthesis was conducted with the QuantiTect Reverse Transcription Kit (Qiagen), following the manufacturer’s protocols.

### 4.3. Library Preparation

Both plasmids pNeae2 and pHEN6 [21,28,41], for bacterial display libraries and periplasmic expression, respectively, were modified by mutagenic PCR in order to exchange the *Sfi*I and *Not*I restriction sites for *Sap*I and *Bsa*I sites, based on the cloning strategy described by [21] for obtaining pNeae2.1 and pHen6.1.

Initial PCR amplification was performed using cDNA from immunized alpaca. The reaction mixture (50 μL) included 1 μL of 10 mM dNTPs, 10 μL of 5x Flexi GoTaq Buffer, 4.5 μL of 25 mM MgCl_2_, 2 μL of 10 μM primer CALL001 (GTCCTGGCTGCTCTTCTACAAGG), 2 μL of 10 μM primer CALL002 (GGTACGTGCTGTTGAACTGTTCC) [42], 0.5 μL of GoTaq (5 U/μL), 2 μL of template DNA, and 28 μL of H_2_O. PCR conditions were as follows: initial denaturation at 94 °C for 5 min, followed by 30 cycles of 94 °C for 1 min, 54 °C for 1 min, and 72 °C for 1 min, with a final extension at 72 °C for 10 min. Products were run on a 1.5% agarose gel to isolate the 700 bp band, which was purified using the GenElute Gel Extraction Kit (Sigma, Darmstadt, Germany), yielding approximately 100–200 ng/μL in 30 μL of preheated H_2_O. The 700 bp purified product was used as a template in a second PCR. The reaction mixture was similar to the first PCR, replacing primers CALL001 and CALL002 with GV145 (AAGCTCTTCATCCTATGGCTCAGGTGCAGCTGGTGG) and GV174 (TTGCTCTTCTTCGGAGGAGACGGTGACCTGGGTC), respectively. PCR conditions were 94 °C for 5 min, followed by 30 cycles of 94 °C for 1 min and 72 °C for 1 min, with a final extension at 72 °C for 10 min. The resulting 400 bp band was isolated, purified as described, and eluted in 30 μL of H_2_O. In a 0.2 mL PCR tube kept on ice, 1.5 μg each of vector pNeae2.1 and insert DNA were combined with 5 μL of 10× T4 Ligase Buffer (NEB, Ipswich, MA, USA), 5 μL of 10× rCutSmart Buffer, 5 μL of *Sap*I (10 U/μL), 0.5 μL of T4 Ligase (2000 U/μL), and H_2_O to a final volume of 100 μL. The reaction was subjected to 40 cycles of 1 min at 37 °C, 1 min at 16 °C, followed by 10 min at 55 °C, 10 min at 80 °C, and held at 4 °C. To the 100 μL reaction, 300 μL of cold 100% ethanol and 10 μL of 3 M sodium acetate (pH 5.2) were added. After a 10 min incubation at *−*20 °C, samples were centrifuged at 20,000× *g* for 30 min at 4 °C, and the pellet was washed with 500 μL of cold 70% ethanol. The pellet was air-dried for 10 min and resuspended in 10 μL of preheated H_2_O at 70 °C. The SOC medium was prewarmed to 37 °C. Electroporation cuvettes (0.1 cm gap) were cooled at −80 °C. Competent cells (50 μL aliquots) were mixed with 2.5–3.3 μL of DNA and electroporated at the EC2 setting on a Bio-Rad Gene Pulser (Bio-Rad Laboratories, Inc., Hercules, CA, USA). Cells were recovered in 1 mL of SOC and incubated at 37 °C with shaking at 200 rpm for 1 h. Transformants were plated on LB agar with chloramphenicol and 2% glucose, incubated overnight at 37 °C, and the library size was estimated based on colony counts from serial dilutions. 

### 4.4. Density Gradient Enrichment

This protocol was carried out according to [27]. NHS-activated Sepharose 4 Fast Flow beads (1 mL) were pre-washed with cold 1 mM HCl and sterile PBS, then incubated overnight with 200 μg of purified protein in PBS with rotation. Unreacted NHS groups were blocked with 0.5 M ethanolamine, and beads were washed with PBS and stored at 4 °C. Library and control bacterial cultures were grown overnight in LB medium with chloramphenicol (25 μg/mL) and 2% glucose or kanamycin (50 μg/mL), respectively. Cultures were then diluted to OD_600nm_ = 0.02 in LB with antibiotic (without glucose) and grown to OD_600nm_ 0.45–0.6 at 37 °C, followed by induction with 50 μM IPTG at 30 °C for 3 h. Both library and control cultures were washed with PBS, adjusted to equal OD, and incubated with 300 μL of protein-coupled beads for 30 min at room temperature. The bead–bacteria mixture was layered onto 6 mL of Ficoll-Paque PLUS (GE Healthcare) in a 15 mL conical tube and centrifuged at 200× *g* for 1 min. The unbound fraction was discarded, and the bead pellet was resuspended in 4 mL of PBS and rotated for 5 min at room temperature. This wash step was repeated six times. After washing, 1 mL of the LB medium was added to the bead suspension and incubated for 5 min at room temperature. Aliquots of 50 μL were plated on LB agar with either 50 μg/mL kanamycin or 25 μg/mL chloramphenicol, both containing 2% glucose. The remaining sample was plated on additional LB chloramphenicol/glucose agar plates. Plates were incubated at 37 °C overnight (20+ h recommended). Colony counts from kanamycin and chloramphenicol first plates were used to assess the specific enrichment of nanobody-expressing bacteria from the library.

### 4.5. Candidate Screening ELISA

Pools of 10 colonies or individual colonies obtained from density gradient separation were inoculated in 2 mL of LB medium and incubated overnight at 37 °C with agitation at 200 rpm. A 100 μL aliquot of the overnight culture was transferred to 1.9 mL of fresh LB medium with 25 μg/mL chloramphenicol and incubated at 37 °C with 200 rpm shaking until reaching an OD_600nm_ of 0.45–0.6. Protein expression was induced by adding IPTG to a final concentration of 50 μM for 3 h at 30 °C with 200 rpm shaking. Cultures were pelleted, resuspended in 1 mL PBS containing 0.2% Triton X-100 (ThermoFisher, Waltham, MA, USA), sonicated on ice for 10 s at 40% amplitude, and centrifuged at 14,000× *g* for 30 min at 4 °C. The supernatant containing the total protein extract was collected for each clone.

Ninety-six-well ELISA plates (Nunc MaxiSorp, ThermoFisher, Waltham, MA, USA) were coated with 100 ng of purified VP2 protein diluted in 1x PBS (pH 7.4) and incubated for 1 h at 37 °C. Plates were washed three times with PBS-T (PBS containing 0.05% Tween-20) for 5 min each and blocked with KPL blocking solution (Seracare) for 30 min at room temperature with gentle shaking. After discarding the blocking solution, each well was incubated with the total protein extract from each clone or pool of clones diluted 1:5 in PBS-T with 5% BSA and incubated for 1 h at room temperature with shaking. Plates were washed three times with PBS-T for 5 min each. The wells were then incubated with Mouse Anti-myc antibody (9B11, Cell Signaling Technology, Danvers, MA, USA) diluted 1:3000 in PBS-T with 5% BSA for 1 h at room temperature, followed by three 5 min washes with PBS-T. Subsequently, Goat Anti-Mouse IgG conjugated to HRP (Invitrogen) at a 1:5000 dilution in PBS-T with 5% BSA was added for 1 h at room temperature, followed by three additional 5 min washes with PBS-T. Signal development was achieved by adding 100 μL of 1-Step Ultra TMB-ELISA substrate (ThermoFisher, Waltham, MA, USA) to each well and incubating for 15 min at 37 °C, then for 5 min at room temperature. Finally, 100 μL of the STOP Solution (ThermoFisher, Waltham, MA, USA) was added, and absorbance was measured at 450 nm using a microplate reader.

### 4.6. Subcloning and Transformation

Nanobody coding sequences were subcloned into the pHEN6.1 vector via a second round of Type IIs enzyme assembly using *Bsa*I (NEB). In a 0.2 mL PCR tube on ice, 1.5 μg each of pHEN6.1 vector and purified plasmid DNA from selected nanobody clones were combined with 5 μL of 10× T4 Ligase Buffer, 5 μL of 10× rCutSmart Buffer, 5 μL of *Bsa*I (10 U/μL), 0.5 μL of T4 Ligase (2000 U/μL), and H_2_O to a final volume of 100 μL. The reaction was cycled 40 times at 37 °C for 1 min and 16 °C for 1 min, followed by 10 min at 55 °C, 10 min at 80 °C, and held at 4 °C. Following assembly, 5 μL of the reaction mixture was transformed into *E. coli* WK6 cells via heat shock at 42 °C. After recovery in 1 mL SOC medium at 37 °C with shaking (200 rpm) for 1 h, transformants were plated on LB agar with chloramphenicol and 2% glucose, and incubated overnight at 37 °C.

### 4.7. Periplasmic Expression and Nanobody Purification

The *E. coli* WK6 strain was used for periplasmic expression using a modified protocol from [28]. A single colony of pHEN6-W25-transformed cells was cultured in 20 mL of LB medium with 100 μg/mL ampicillin and 1% glucose at 37 °C with agitation for 16 h. This pre-culture was diluted into 1 L of Terrific Broth (TB) containing 100 μg/mL ampicillin, 2 mM MgCl_2_, and 0.1% glucose, and grown at 37 °C to an OD_nm_ of 0.6–0.9. Nanobody expression was induced by adding 1 mM IPTG, followed by incubation at 28 °C for 20 h. Bacteria were harvested by centrifugation at 8000 rpm for 8 min at 4 °C. The bacterial pellet was resuspended in 12 mL TES buffer (0.2 M Tris, 0.5 mM EDTA, 0.5 M sucrose, pH 8.0) and incubated on ice for 1 h. An additional 18 mL of TES buffer was added, and the suspension was further incubated on ice for 1 h. Cell debris was pelleted by centrifugation at 8000 rpm at 4 °C. The supernatant was applied to a 5 mL HisPur Ni–NTA agarose resin pre-equilibrated with binding buffer (50 mM Tris, 500 mM NaCl, 10 mM imidazole, and pH 7.5). Bound proteins were washed with binding buffer (50 mM Tris, 500 mM NaCl, 10 mM imidazole, and pH 7.5) and eluted in 15 mL of elution buffer (50 mM Tris, 150 mM NaCl, 150 mM imidazole, 1 mM DTT, and pH 7.5). Purified Nanobodies were analyzed by SDS-PAGE to confirm purity and integrity.

### 4.8. Dot Blot Analysis

The total protein extract from the IPNv strain VR-299 was used as the antigen, and serial dilutions ranging from 125.0 to 2.0 µg/mL were prepared. Proteins were immobilized by air-drying the nitrocellulose membrane for 30 min at room temperature. Non-specific binding sites were blocked with blocking buffer (PBS containing 0.1% Tween-20 and 5% bovine serum albumin, BSA) for 30 min at room temperature with gentle agitation. After discarding the blocking solution, membranes were incubated for 1 h at room temperature with agitation using either Nb P9 or anti-VP2 antibody (clone 1B3/E10; Ango) at a 1:1000 dilution in PBS-T supplemented with 5% BSA. This was followed by three washes (5 min each) with PBS-T.

For Nb P9 detection, membranes were further incubated with a mouse anti-Myc antibody (clone 9B11, Cell Signaling, Danvers, MA, USA) diluted at 1:3000 in PBS-T containing 5% BSA for 1 h at room temperature, followed by three washes with PBS-T. Subsequently, membranes were incubated with a goat anti-mouse IgG conjugated to HRP (Invitrogen Waltham, Massachusetts, USA) diluted at 1:5000 in PBS-T containing 5% BSA for 1 h at room temperature, followed by three additional washes with PBS-T. Signal detection was performed using ECL reagent (ThermoFisher, Waltham, MA, USA).

### 4.9. Structure Prediction

The structural prediction of the P9 nanobody was performed using AlphaFold, a machine learning model for protein structure prediction. The amino acid sequence of P9, including annotated complementarity-determining regions (CDRs), was input into the AlphaFold Protein Structure Database. AlphaFold-generated models were analyzed to confirm the overall folding and structural integrity, focusing particularly on the spatial orientation of CDR loops, which are critical for antigen recognition [43]. The resulting structure was visualized and annotated to highlight CDRs, facilitating further insights into the binding potential of P9 against the IPNv VP2 protein.

### 4.10. Molecular Docking

The amino-acid sequences of the viral protein VP2 and the host protein P9 were used as inputs for structure-based interaction modelling. Complex models were generated with two independent predictors, AlphaFold 3 and the Boltz-1 docking workflow, to minimize method-specific bias [44,45]. Each run produced a quaternary structure for the VP2–P9 heterodimer and, for benchmarking purposes, for a positive control pair that is known to interact (UniProt IDs O95400–P06729) and a negative control pair that does not (O95400–P55036).

Model quality and interface confidence were evaluated with five complementary metrics: ipTM, ipSAE, pDockQ, pDockQ2, and LIS [45,46,47]. ipTM measures the agreement of the predicted interface with a reference TM-score, but can be deflated by flexible or non-interacting regions. ipSAE refines ipTM by focusing only on high-confidence residue pairs within the interface, thereby avoiding penalties from disordered segments. pDockQ and its updated version pDockQ2 integrate per-residue confidence (pLDDT) and predicted aligned error (PAE) to yield an interface-specific reliability score. LIS provides an orthogonal ligand-interface score produced by the Boltz-1 workflow.

To visualize potential contact patches, ParaSurf 1.3 was applied to every model. The software assigns, for each residue and atom, a probability of engaging in antigen–antibody-like surface interactions; values > 0.5 were considered high-probability hotspots [48]. All structural figures were rendered in ChimeraX v1.10.

### 4.11. Cell Culture

The salmon head kidney–derived cell line SHK-1 (ECACC 97111106) was maintained in Leibovitz’s L-15 medium (Thermo Fisher Scientific, Waltham, MA, USA) supplemented with 10% (*v*/*v*) certified heat-inactivated fetal bovine serum (FBS; Cytiva, Marlborough, MA, USA). Cultures were incubated at 21 °C under atmospheric conditions, as the L-15 medium is buffered for CO_2_-independent growth. Cells were routinely passaged upon reaching 80–90% confluence using enzymatic dissociation with trypsin-EDTA (ThermoFisher, Waltham, MA, USA, according to experimental needs. For immunofluorescence assays, monolayers were subsequently infected with the infectious pancreatic necrosis virus (IPNv). Routine screening for mycoplasma contamination was performed by PCR using specific primers.

### 4.12. In Vitro IPNv Infection

The IPNv strain VR-299 was used in this study. Viral stocks were propagated and titrated in SHK-1 (ECACC 97111106) using the tissue culture infectious dose 50% (TCID_50_) method. Virus preparations were aliquoted and stored at −80 °C until further use. For in vitro infection assays, SHK-1 cell monolayers were infected with IPNV VR-299 at a multiplicity of infection (MOI) of 5. The virus was adsorbed for 1 h at 15 °C in a minimal volume of L-15 medium containing 2% FBS, with gentle agitation every 15 min. After adsorption, the inoculum was aspirated, cells were washed twice with PBS, and fresh complete medium was added. Infected and mock-infected cultures were incubated for 36 h post-infection (hpi) before downstream analyses.

### 4.13. Immunofluorescence

Imprints of *Salmo salar* head kidney of IPNv-infected and healthy individuals, tested by qPCR, were fixed with methanol for 30 min and then washed three times with PBS. SHK-1 infected and uninfected cells were fixed with Paraformaldehyde 3.7% and then washed three times with PBS. Samples were incubated for 45 min at 37 °C with purified nanobodies. Cells were then washed three times in PBS tween, followed by incubation with a mouse anti-myc antibody (1:3000, Cell Signaling, Danvers, MA, USA) for 45 min at 37 °C. After three PBS washes, an anti-mouse Alexa 647 or Alexa 488 secondary antibody was applied for 30 min at 37 °C. Nuclei were stained with 0.1 mg/mL DAPI for 10 min at room temperature. After a final PBS wash, samples were mounted using ProLong™ Gold Antifade Mountant (ThermoFisher, Waltham, MA, USA). Images were acquired using a Celldiscoverer 7 high-content automatic microscope (Carl Zeiss GmbH, Jena, Germany).

## 5. Conclusions

In this work, we present a streamlined pipeline for the rapid discovery and development of nanobodies against the VP2 protein of IPNv, built upon a novel Type IIS-driven assembly method for library generation. This approach simplifies the construction of highly diverse nanobody libraries. A key strength of the pipeline is its adaptability, as it can be rapidly reconfigured to target virtually any protein of interest, including antigens from other aquaculture pathogens as well as those from human or other animal diseases. By markedly reducing the time required for nanobody discovery, this method provides a powerful tool to accelerate both fundamental research and the development of diagnostic and therapeutic applications, ultimately contributing to improved disease management in aquaculture.

## Data Availability

The data presented in this study are available on request from the corresponding author.

## Supplementary Materials

The following supporting information can be downloaded at: https://www.mdpi.com/article/10.3390/ijms26199350/s1.

## Abbreviations

The following abbreviations are used in this manuscript:

IPNv	Infectious pancreatic necrosis virus
Nb	Nanobody
CDR	complementarity-determining regions

## References

[1] Dobos P. (1995). The molecular biology of infectious pancreatic necrosis virus (IPNV). Annu. Rev. Fish Dis..

[2] Azad A.A., Jagadish M.N., Brown M.A., Hudson P.J. (1987). Deletion mapping and expression in Escherichia coli of the large genomic segment of a birnavirus. Virology.

[3] Håvarstein L.S., Kalland K.H., Christie K.E., Endresen C. (1990). Sequence of the large double-stranded RNA segment of the N1 strain of infectious pancreatic necrosis virus: A comparison with other Birnaviridae. J. Gen. Virol..

[4] Coulibaly F., Chevalier C., Gutsche I., Pous J., Navaza J., Bressanelli S., Delmas B., Rey F.A. (2005). The birnavirus crystal structure reveals structural relationships among icosahedral viruses. Cell.

[5] Coulibaly F., Chevalier C., Delmas B., Rey F.A. (2010). Crystal structure of an Aquabirnavirus particle: Insights into antigenic diversity and virulence determinism. J. Virol..

[6] Caswell-Reno P., Reno P.W., Nicholson B.L. (1986). Monoclonal antibodies to infectious pancreatic necrosis virus: Analysis of viral epitopes and comparison of different isolates. J. Gen. Virol..

[7] Asche F., Bjørndal T. (2011). The Economics of Salmon Aquaculture.

[8] Smail D.A., Munro E.S., Roberts R.J. (2012). The virology of teleosts. Fish Pathology.

[9] Labraña R., Espinoza J.C., Kuznar J. (2008). Detección del virus de la necrosis pancreática infecciosa (IPNV) en sedimentos de agua dulce. Arch. Med. Vet..

[10] Manríquez R.A., Sandoval M., Loncoman C., Tafalla C., Avendaño-Herrera R., Cárcamo J.G. (2023). Epigenetic reprogramming around IFN1 and IFNy2 promoters in rainbow trout cells inoculated with infectious pancreatic necrosis virus (IPNV). Fish Shellfish. Immunol..

[11] Godoy M., Kibenge M.J.T., Montes de Oca M., Pontigo J.P., Coca Y., Caro D., Kusch K., Suarez R., Burbulis I., Kibenge F.S.B. (2022). Isolation of a New Infectious Pancreatic Necrosis Virus (IPNV) Variant from Genetically Resistant Farmed Atlantic Salmon (*Salmo salar*) during 2021–2022. Pathogens.

[12] Hillestad B., Johannessen S., Melingen G.O., Moghadam H.K. (2021). Identification of a New Infectious Pancreatic Necrosis Virus (IPNV) Variant in Atlantic Salmon (*Salmo salar* L.) that can Cause High Mortality Even in Genetically Resistant Fish. Front. Genet..

[13] Jovčevska I., Muyldermans S. (2020). The therapeutic potential of nanobodies. BioDrugs.

[14] Wang J., Kang G., Yuan H., Cao X., Huang H., de Marco A. (2021). Research progress and applications of multivalent, multispecific and modified nanobodies for disease treatment. Front. Immunol..

[15] Casini A., Storch M., Baldwin G.S., Ellis T. (2015). Bricks and blueprints: Methods and standards for DNA assembly. Nat. Rev. Mol. Cell Biol..

[16] Engler C., Youles M., Gruetzner R., Ehnert T.-M., Werner S., Jones J.D.G., Patron N.J., Marillonnet S. (2014). A golden gate modular cloning toolbox for plants. ACS Synth. Biol..

[17] Sarrion-Perdigones A., Vazquez-Vilar M., Palací J., Castelijns B., Forment J., Ziarsolo P., Blanca J., Granell A., Orzaez D. (2013). GoldenBraid 2.0: A comprehensive DNA assembly framework for plant synthetic biology. Plant Physiol..

[18] Ellis T., Adie T., Baldwin G.S. (2011). DNA assembly for synthetic biology: From parts to pathways and beyond. Integr. Biol..

[19] Engler C., Kandzia R., Marillonnet S. (2008). A one pot, one step, precision cloning method with high throughput capability. PLoS ONE.

[20] Andreou A.I., Nakayama N. (2018). Mobius Assembly: A versatile Golden-Gate framework towards universal DNA assembly. PLoS ONE.

[21] Pollak B., Matute T., Nuñez I., Cerda A., Lopez C., Vargas V., Kan A., Bielinski V., von Dassow P., Dupont C.L. (2020). Universal loop assembly: Open, efficient and cross-kingdom DNA fabrication. Synth. Biol..

[22] Halleran A.D., Swaminathan A., Murray R.M. (2018). Single Day Construction of Multigene Circuits with 3G Assembly. ACS Synth. Biol..

[23] Iverson S.V., Haddock T.L., Beal J., Densmore D.M. (2016). CIDAR MoClo: Improved MoClo Assembly Standard and New *E. coli* Part Library Enable Rapid Combinatorial Design for Synthetic and Traditional Biology. ACS Synth. Biol..

[24] Lin D., O’Callaghan C.A. (2018). MetClo: Methylase-assisted hierarchical DNA assembly using a single type IIS restriction enzyme. Nucleic Acids Res..

[25] Lebendiker M., Danieli T. (2014). Production of prone-to-aggregate proteins. FEBS Lett..

[26] Zou Z., Cao L., Zhou P., Su Y., Sun Y., Li W. (2008). Hyper-acidic protein fusion partners improve solubility and assist correct folding of recombinant proteins expressed in Escherichia coli. J. Biotechnol..

[27] Valenzuela Nieto G., Jara R., Watterson D., Modhiran N., Amarilla A.A., Himelreichs J., Khromykh A.A., Salinas-Rebolledo C., Pinto T., Cheuquemilla Y. (2021). Potent neutralization of clinical isolates of SARS-CoV-2 D614 and G614 variants by a monomeric, sub-nanomolar affinity nanobody. Sci. Rep..

[28] Salema V., Marín E., Martínez-Arteaga R., Ruano-Gallego D., Fraile S., Margolles Y., Teira X., Gutierrez C., Bodelón G., Fernández L.Á. (2013). Selection of single domain antibodies from immune libraries displayed on the surface of, *E. coli* cells with two β-domains of opposite topologies. PLoS ONE.

[29] Brochet X., Lefranc M.-P., Giudicelli V. (2008). IMGT/V-QUEST: The highly customized and integrated system for IG and TR standardized V-J and V-D-J sequence analysis. Nucleic Acids Res..

[30] Abdullah A., Olsen C.M., Hodneland K., Rimstad E. (2015). A polyprotein-expressing salmonid alphavirus replicon induces modest protection in atlantic salmon (*Salmo salar*) against infectious pancreatic necrosis. Viruses.

[31] Levican J., Miranda-Cárdenas C., Soto-Rifo R., Aguayo F., Gaggero A., León O. (2017). Infectious pancreatic necrosis virus enters CHSE-214 cells via macropinocytosis. Sci. Rep..

[32] Isaacs A., Nieto G.V., Zhang X., Modhiran N., Barr J., Thakur N., Low Y.S., Parry R.H., Barnes J.B., Jara R. (2025). A nanobody-based therapeutic targeting Nipah virus limits viral escape. Nat. Struct. Mol. Biol..

[33] Valenzuela-Nieto G., Miranda-Chacon Z., Salinas-Rebolledo C., Jara R., Cuevas A., Berking A., Rojas-Fernandez A. (2022). Nanobodies: COVID-19 and Future Perspectives. Front. Drug Discov..

[34] Modhiran N., Lauer S.M., Amarilla A.A., Hewins P., Lopes van den Broek S.I., Low Y.S., Thakur N., Liang B., Nieto G.V., Jung J. (2023). A nanobody recognizes a unique conserved epitope and potently neutralizes SARS-CoV-2 omicron variants. Iscience.

[35] Wu X., Cheng L., Fu M., Huang B., Zhu L., Xu S., Shi H., Zhang D., Yuan H., Nawaz W. (2021). A potent bispecific nanobody protects hACE2 mice against SARS-CoV-2 infection via intranasal administration. Cell Rep..

[36] Li T., Cai H., Yao H., Zhou B., Zhang N., van Vlissingen M.F., Kuiken T., Han W., GeurtsvanKessel C.H., Gong Y. (2021). A synthetic nanobody targeting RBD protects hamsters from SARS-CoV-2 infection. Nat. Commun..

[37] Hanke L., Vidakovics Perez L., Sheward D.J., Das H., Schulte T., Moliner-Morro A., Corcoran M., Achour A., Karlsson Hedestam G.B., Hällberg B.M. (2020). An alpaca nanobody neutralizes SARS-CoV-2 by blocking receptor interaction. Nat. Commun..

[38] Romao E., Morales-Yanez F., Hu Y., Crauwels M., De Pauw P., Hassanzadeh G.G., Devoogdt N., Ackaert C., Vincke C., Muyldermans S. (2016). Identification of Useful Nanobodies by Phage Display of Immune Single Domain Libraries Derived from Camelid Heavy Chain Antibodies. Curr. Pharm. Des..

[39] Sommerset I., Krossøy B., Biering E., Frost P. (2005). Vaccines for fish in aquaculture. Expert Rev. Vaccines.

[40] Hassanzadeh-Ghassabeh G., Devoogdt N., De Pauw P., Vincke C., Muyldermans S. (2013). Nanobodies and their potential applications. Nanomedicine.

[41] Salema V., López-Guajardo A., Gutierrez C., Mencía M., Fernández L.Á. (2016). Characterization of nanobodies binding human fibrinogen selected by, *E. coli* display. J. Biotechnol..

[42] Els Conrath K., Lauwereys M., Wyns L., Muyldermans S. (2001). Camel single-domain antibodies as modular building units in bispecific and bivalent antibody constructs. J. Biol. Chem..

[43] Jumper J., Evans R., Pritzel A., Green T., Figurnov M., Ronneberger O., Tunyasuvunakool K., Bates R., Žídek A., Potapenko A. (2021). Highly accurate protein structure prediction with AlphaFold. Nature.

[44] Wohlwend J., Corso G., Passaro S., Getz N., Reveiz M., Leidal K., Swiderski W., Atkinson L., Portnoi T., Chinn I. (2025). Boltz-1 Democratizing Biomolecular Interaction Modeling. BioRxiv.

[45] Abramson J., Adler J., Dunger J., Evans R., Green T., Pritzel A., Ronneberger O., Willmore L., Ballard A.J., Bambrick J. (2024). Accurate structure prediction of biomolecular interactions with AlphaFold 3. Nature.

[46] Bryant P., Pozzati G., Elofsson A. (2022). Improved prediction of protein-protein interactions using AlphaFold2. Nat. Commun..

[47] Basu S., Wallner B. (2016). DockQ: A Quality Measure for Protein-Protein Docking Models. PLoS ONE.

[48] Papadopoulos A.-M., Axenopoulos A., Iatrou A., Stamatopoulos K., Alvarez F., Daras P. (2025). ParaSurf: A surface-based deep learning approach for paratope-antigen interaction prediction. Bioinformatics.

